# Supplementary material for: High-fat diet and estrogen modulate the gut microbiota in a sex-dependent manner in mice
Source: Commun Biol. 2023 Jan 9;6:20. doi: 10.1038/s42003-022-04406-5 (PMC9829864; doi:10.1038/s42003-022-04406-5)
Supplement: Supplementary file 2 — Supplementary figures and tables [file 42003_2022_4406_MOESM2_ESM.pdf]

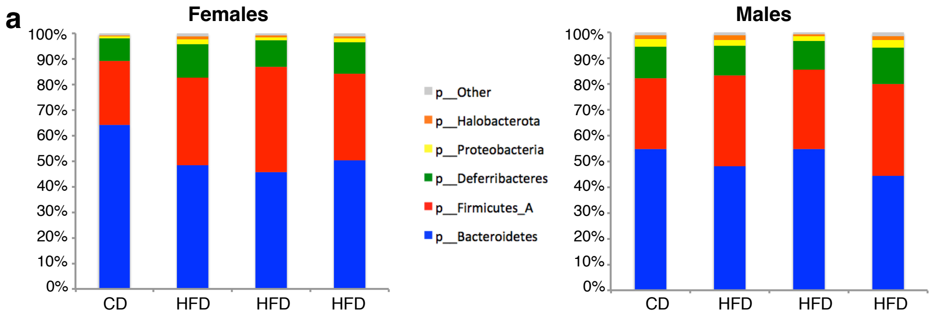

b

	CD F	HFD F	E2 F	DPN F	CD M	HFD M	E2 M	DPN M
p__Bacteroidetes	64.23**	48.44	45.70	50.38	54.71	48.12	54.75	44.42
p__Firmicutes_A	24.98	34.41	#41.34	33.88	27.53	35.25	30.84	35.47
p__Deferribactarota	8.93	12.74	10.12	12.24	12.33	11.36	11.11	14.10
p__Proteobacteria	# 0.72	1.93	1.28	1.52	2.66	2.44	1.63	3.00
p__Halobacterota	# 0.45	1.33	0.77	0.94	1.61	1.61	0.84	1.68
p__Other	0.69	1.15	0.78	1.04	1.16	1.21	0.82	1.33
Firmicutes/Bacteroidetes	0.40	0.75	# 1.18	0.73	0.53	0.78	0.64	0.90

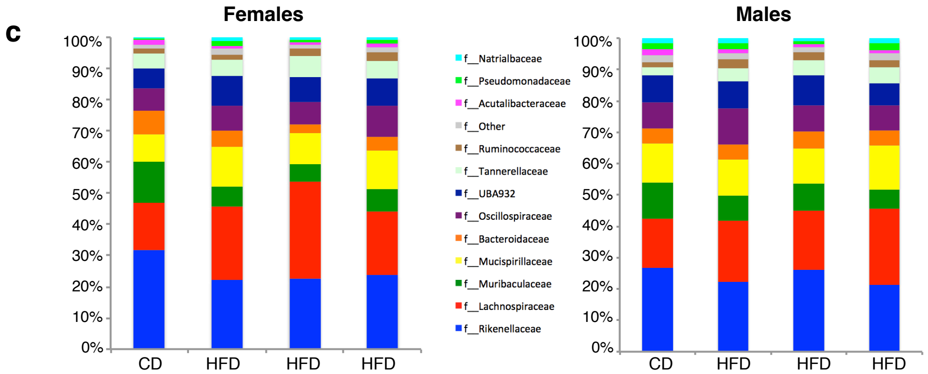

d

	CD F	HFD F	E2 F	DPN F	CD M	HFD M	E2 M	DPN M
f__Rikenellaceae	31.85 **	22.08	22.75	23.63	26.75	22.26	26.24	21.20
f__Lachnospiraceae	14.89	23.55	#31.12	20.43	15.75	19.39	18.82	24.39
f__Muribaculaceae	13.27***	6.54	#5.33	7.40	11.45 *	8.21	8.65	6.17
f__Mucispirillaceae	8.93	12.74	10.12	12.24	12.33	11.36	11.11	14.10
f__Bacteroidaceae	7.48	5.04	2.82	4.50	5.00	4.84	5.37	4.61
f__Oscillospiraceae	7.12	#8.14	7.27	9.70	8.12**	11.67	8.43**	8.11**
f__UBA932	#6.42**	9.50	8.00	9.00	8.70	8.41	9.44	7.14
f__Tannerellaceae	#5.00	5.10	6.69	5.69	2.66	4.27	4.89	5.15
f__Ruminococcaceae	1.51	1.83	2.15	#2.54 *	1.56 ***	2.77	2.55	1.98 *
f__Other	1.28	1.91	1.31	1.70	2.16	2.12	1.56	2.40
f__Acutalibacteraceae	#1.26 ***	#0.77	0.70	1.07	1.97	1.28	0.93	0.89
f__Pseudomonadaceae	#0.53	1.47	0.97	1.14	1.96	1.82	1.16	2.17
f__Natrialbaceae	#0.45	1.33	0.77	0.94	1.61	1.61	0.84	1.68

Supplementary Figure 1: Effect of sex, HFD, and estrogenic ligands on the abundance of different phyla and families. (a) Taxonomy plots of the relative abundance of the most abundant phyla in both females and males, along with **(b)** corresponding statistics. **(c)** Taxonomy plots of the relative abundance of the most abundant families in both females and males, along with **(d)** corresponding statistics. One-way ANOVA with uncorrected Fisher's Least Significant Difference (LSD) test was used. *Indicates significant difference compared to HFD + vehicle, $^{*}p < 0.05$; $^{**}p < 0.01$; $^{***}p < 0.001$. $^{\#}$ Indicates significant sex difference. N=64 (n=8, per group).

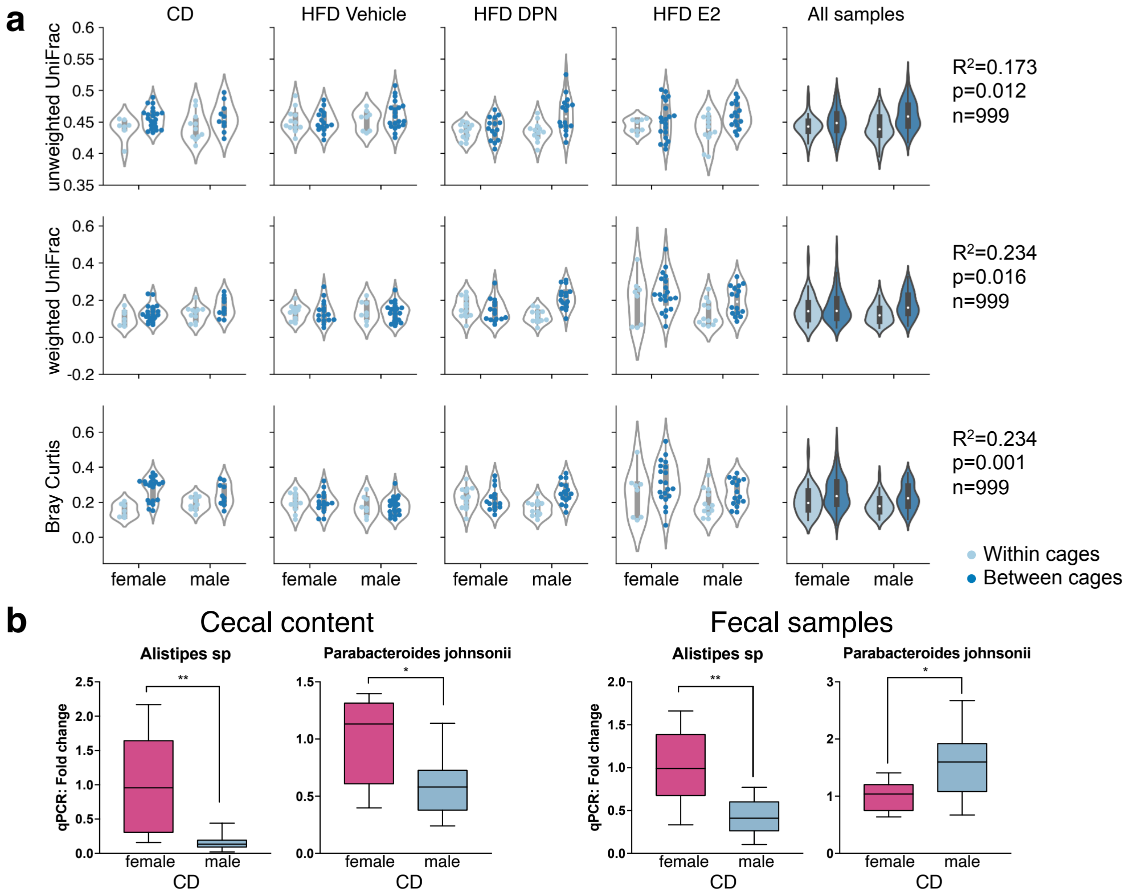

Supplementary Figure 2: Mice in the same cage are more similar to each other (within cage effect) compared to mice in other cages (between cage effect) of the same sex and undergoing the same treatment. (a) Violin plots show the dissimilarity of microbiomes of mice in the same cage (left, light blue) compared to between cages (right, blue). A higher value indicates a greater dissimilarity between the animals. This is shown for all three metrics: unweighted UniFrac (top), weighted UniFrac (middle), and Bray-Curtis distance for both females and males. N=64 (n=8 per group, except males CD n=7). **(b)** qPCR analysis demonstrating sex differences in the abundance of *Alistipes sp* and *Parabacteroides johnsonii* during CD, in both cecal (n=11 females, n=9 males) and fecal samples (n=10 per sex). The boxplots are shown as median (line), interquartile range (box), and minimum to maximum data range (whisker). Student's two-tailed t-test, $^{*}p < 0.05$; $^{**}p < 0.01$.

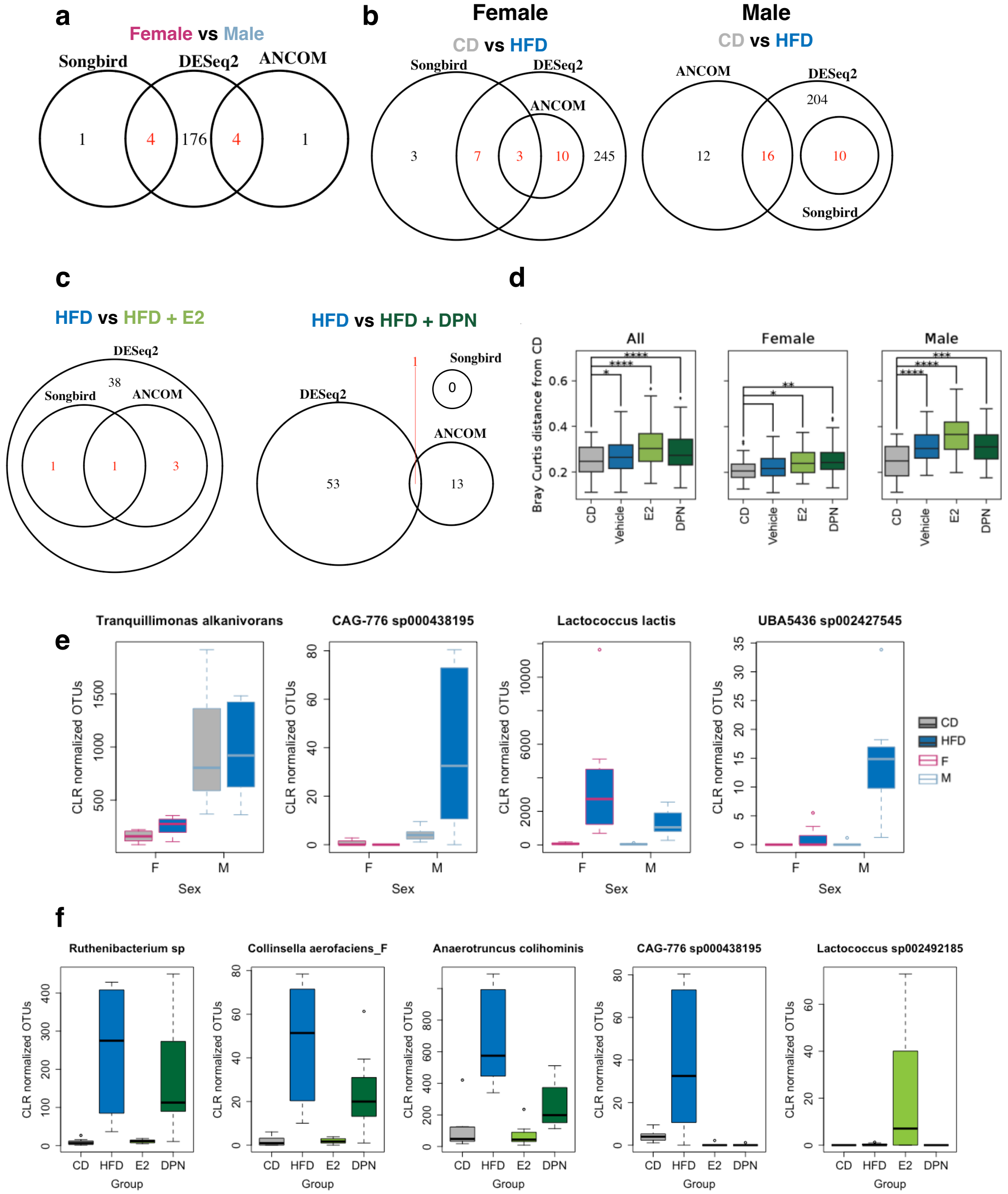

Supplementary Figure 3: DESeq2 and ANCOM-II identify additional significantly altered species. Venn diagram showing the number and overlap of differentially expressed species generated by Songbird, DESeq2, and ANCOM-II between **(a)** sexes, **(b)** diet (HFD), and **(c)** estrogenic ligand treatment in males (E2 and DPN). **(d)** Boxplot showing the Bray-Curtis distance from CD. **(e)** Boxplots of centered log-ratio (CLR) normalized operational taxonomic unit (OTU) counts for the four additional differentially abundant species between the sexes identified with both DESeq2 and ANCOM-II. **(f)** Boxplots of CLR normalized OTU counts for the differentially abundant species upon ligands treatments in males. N=64 (n=8 per group, except males CD n=7). The boxplots are shown as median (line), interquartile range (box), and minimum to maximum data range (whisker). *FDR<0.05; **FDR<0.01; ***FDR<0.001; **** FDR<0.0001 (Benjamini-Hochberg-adjusted p-value).

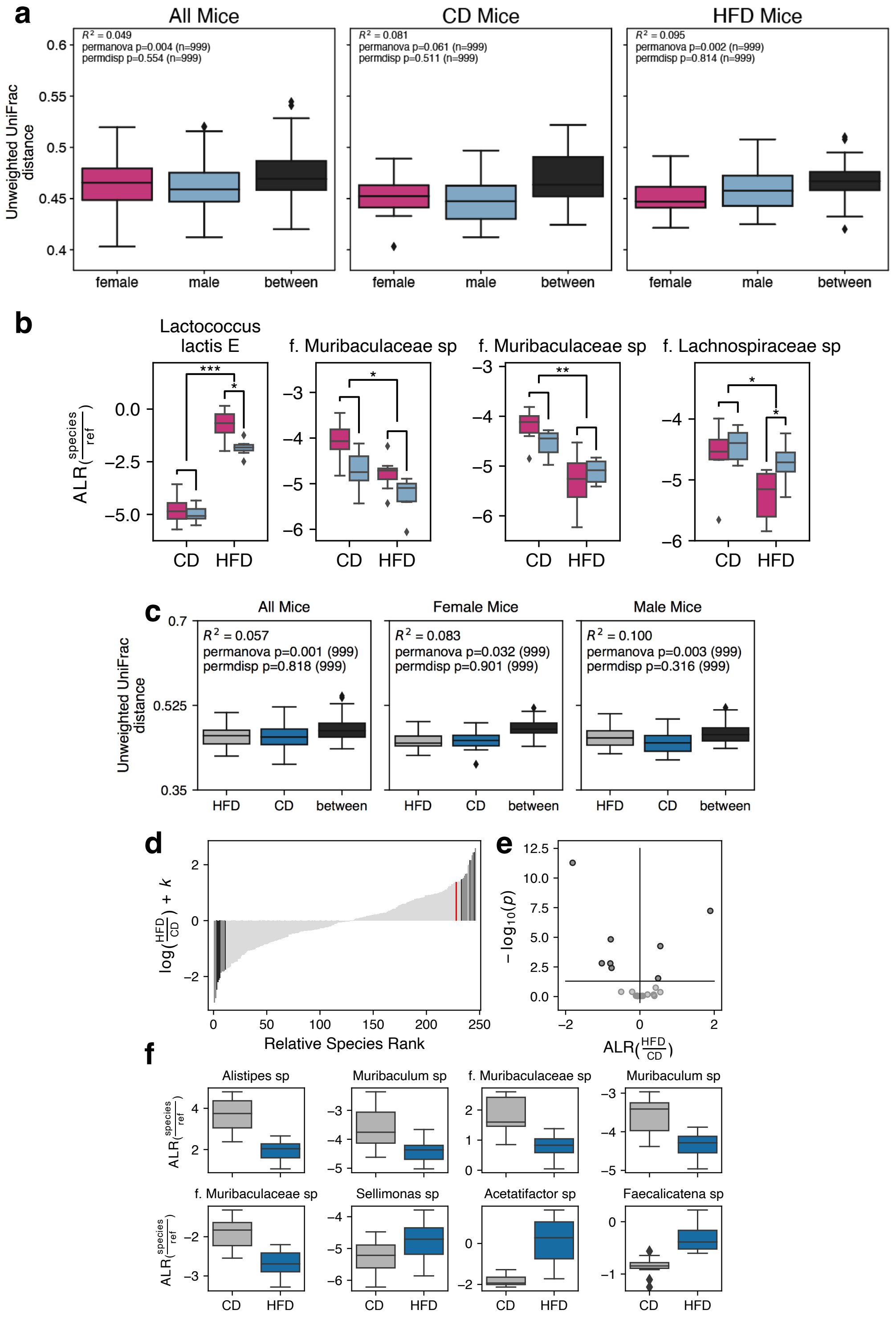

Supplementary Figure 4: Significant sex differences during HFD identified using a sex interaction model. (a) Boxplots presenting the beta diversity per sex, with unweighted UniFrac distance. **(b)** Boxplot of the additive-log-ratio (ALR) transformed taxa showing the significantly altered species upon HFD with a sex-interaction model. **(c)** Boxplots presenting the beta diversity per diet, with unweighted UniFrac distance. **(d)** Histogram plotting the species rank against the log fold change difference between CD and HFD, both sexes combined. **(e)** Volcano plot showing the significantly altered species (HFD vs CD), both sexes combined. **(f)** Boxplot of the ALR-transformed taxa showing the species significantly altered by HFD, both sexes combined. N=64 (n=8 per group, except males CD n=7). The boxplots are shown as median (line), interquartile range (box), and minimum to maximum data range (whisker). *FDR<0.05; **FDR<0.01; ***FDR<0.001 (Benjamini-Hochberg-adjusted p-value).

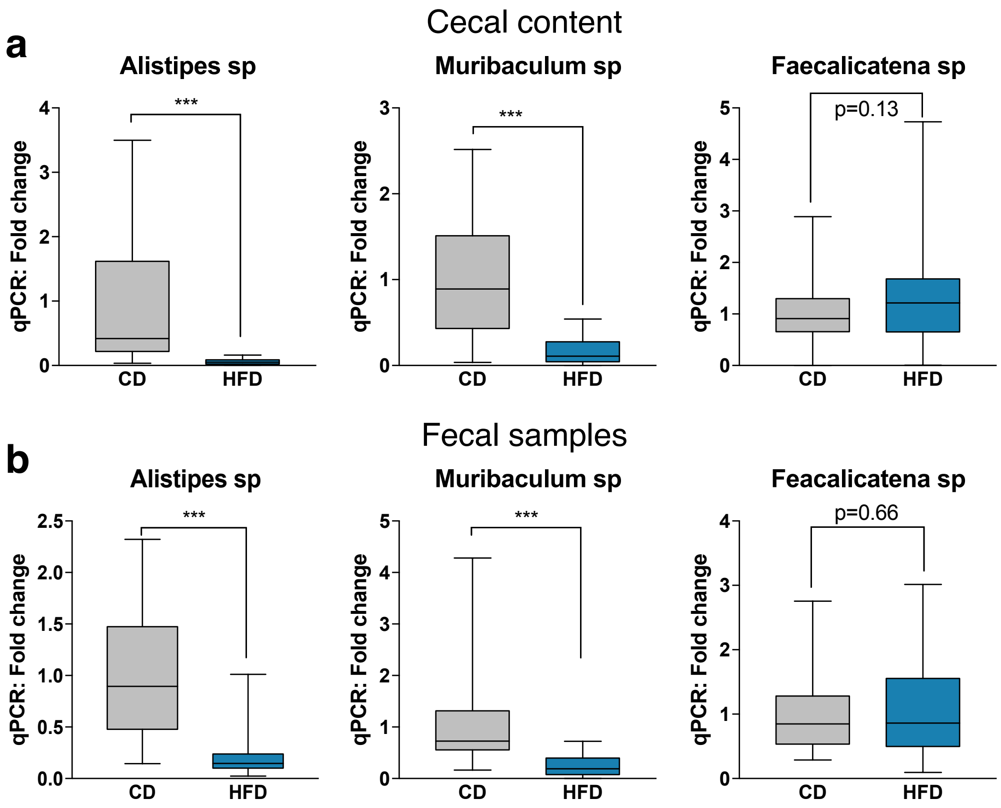

Supplementary Figure 5: HFD-induced changes in the cecal microbiome confirmed by qPCR in cecal and fecal samples. qPCR confirmation of HFD-induced changes identified by WGS (cecal microbiome) for *Alistipes* sp, *Muribaculum* and *Faecalicatena* genera in **(a)** cecal content (n=20 CD, n=24 HFD, sexes combined) and **(b)** fecal samples (n=20 CD, n=24 HFD, sexes combined). The boxplots are shown as median (line), interquartile range (box), and minimum to maximum data range (whisker). Two-tailed Student's t-test, $^{***}p < 0.001$.

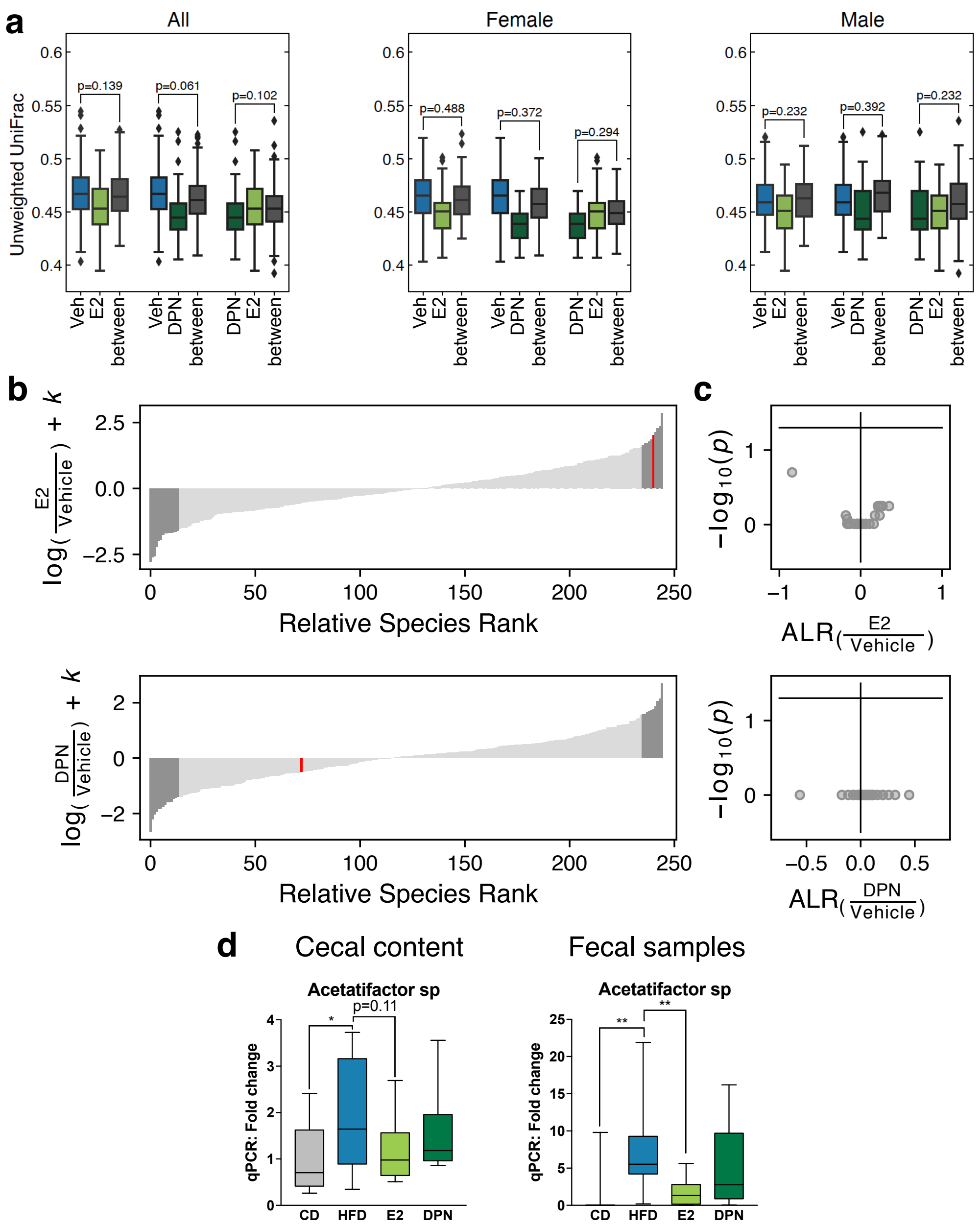

Supplementary Figure 6: Estrogenic treatment does not have a significant impact on the cecal microbiome when the sexes are combined. (a) Boxplots showing the unweighted UniFrac distance between vehicle, E2, and DPN treatment in sexes combined (left) and separated (right), during HFD. **(b)** Histogram plots of the species rank against the log fold change difference (E2 vs vehicle, and DPN vs vehicle), adjusted for sex. **(c)** Volcano plot showing the significant differentially abundant species (E2 vs vehicle, and DPN vs vehicle), adjusted for sex. **(d)** qPCR confirmation of E2 regulation of *Acetatifactor sp* in cecal and fecal samples, in males (n=9 for CD, n=14 for HFD, n=8 for E2, and n=8 for DPN, with two outliers identified and removed for both E2 and DPN treatments). The boxplots are shown as median (line), interquartile range (box), and minimum to maximum data range (whisker). Student's two-tailed t-test, *$p < 0.05$; **$p < 0.01$.

Songbird / DESeq2 / ANCOM-II	Songbird / DESeq2	DESeq2 / ANCOM-II
s__Turicibacter sp002311155	s__CAG-873 sp002490635	s__Lactococcus lactis_E
s__Brachyspira hampsonii_B	s__Muribaculum sp002358615	s__Alistipes sp002428825
s__CAG-552 sp000435495	s__UBA7597 sp002474405	s__Helicobacter_D sp002297555
	s__CAG-115 sp002492075	s__Enterococcus_B durans
	s__Ruthenibacterium lactatiformans	s__Turicibacter sp002493395
	s__Eubacterium_E sp900016875	s__Ruthenibacterium sp003149955
	s__Blautia sp000432195	s__Turicibacter sanguinis
		s__NK4A144 sp000622025
		s__Phoenicibacter massiliensis
		s__CAG-269 sp000437215

Supplementary Table 1: Species identified as differentially abundant upon diet (CD vs HFD) in females with at least two methods (DESeq2, Songbird, and/or ANCOM-II).

Songbird / DESeq2	ANCOM-II / DESeq2
s__Turicibacter sp002311155	s__Lactococcus lactis_E
s__ER4 sp000765235	s__Acetatifactor sp002490995
s__Ruthenibacterium sp002315015	s__UBA7173 sp001689685
s__Collinsella aerofaciens_F	s__Muribaculum sp002492595
s__Brachyspira hampsonii_B	s__CAG-475 sp900322215
s__NK3B98 sp000421005	s__Dorea sp002492335
s__CAG-110 sp002372735	s__Streptococcus orisratti
s__Agathobaculum butyriciproducens	s__Megasphaera sp900066485
s__CAG-485 sp002491165	s__Phoenicibacter massiliensis
s__UBA7597 sp002474405	s__UBA3206 sp002363705
	s__Enterococcus_B durans
	s__UBA5436 sp002427545
	s__CAG-1138 sp000434675
	s__UBA2897 sp002350105
	s__Turicibacter sp002493395
	s__Turicibacter sanguinis

Supplementary Table 2: Species identified as differentially abundant upon diet (CD vs HFD) in males, with two methods (DESeq2 and Songbird or ANCOM-II)

	Female		Male	
Diet/Treatment	BW (g)	SEM	BW (g)	SEM
CD	22.19	0.63	29.89	1.20
HFD/Veh	30.54	1.24	43.58	0.73
HFD/E2	29.82	1.23	45.19	0.94
HFD/DPN	31.89	1.21	41.85	1.99

Supplementary Table 3: Average body weight per group (N=64, n=8 per group) at end of the experiment.

Primer	FW primer (5'-3')	RV primer (5'-3')
Acetatifactor sp002490995	AATACGCGCAGGAGTATGGG	GGTTGCCGTGAAAAGAACCC
Alistipes sp002428825	GGGTTGCACGAGGTTCTTTC	GTTTCGTTTCTCTGTGGGAGC
Faecalicatena genus	GGCGTGCTTAACACATGCAA	CCGCTCAGTCACAACACTCT
Muribaculum genus	AGGGTGTAGCTCTACGGAGT	ACTTAAGCCGACACCTCACG
Parabacteroides johnsonii	GTAGTCAGACCGACCTTGAAAGAG	CTTAAGCCGACACCTCACGG
Total bacteria	CGGCAACGAGCGCAACCC	CCATTGTAGCACGTGTGTAGCC

Supplementary Table 4: Primer sequences
