# Supplementary material for: High-fat diet and estrogen modulate the gut microbiota in a sex-dependent manner in mice
Source: Commun Biol. 2023 Jan 9;6:20. doi: 10.1038/s42003-022-04406-5 (PMC9829864; doi:10.1038/s42003-022-04406-5)
Supplement: Supplementary file 3 — Description of Additional Supplementary Data [file 42003_2022_4406_MOESM3_ESM.docx]

**Description of Additional Supplementary Files**

**File name:** Supplemental Data 1 and Supplemental Data 2

**Description:** The source data for the tables illustrated in the graphs in the paper is provided in qza format for use in the software packet QIIME2
