# Supplementary material for: High-fat diet and estrogen modulate the gut microbiota in a sex-dependent manner in mice
Source: Commun Biol. 2023 Jan 9;6:20. doi: 10.1038/s42003-022-04406-5 (PMC9829864; doi:10.1038/s42003-022-04406-5)
Supplement: Supplementary file 6 — Reporting Summary-New [file 42003_2022_4406_MOESM6_ESM.pdf]

nature portfolio

Corresponding author(s): Cecilia Williams
Last updated by author(s): Dec 8, 2022

Reporting Summary

Nature Portfolio wishes to improve the reproducibility of the work that we publish. This form provides structure for consistency and transparency in reporting. For further information on Nature Portfolio policies, see our Editorial Policies and the Editorial Policy Checklist.

Statistics

For all statistical analyses, confirm that the following items are present in the figure legend, table legend, main text, or Methods section.

n/a	Confirmed	
☐	☒	The exact sample size (*n*) for each experimental group/condition, given as a discrete number and unit of measurement
☐	☒	A statement on whether measurements were taken from distinct samples or whether the same sample was measured repeatedly
☐	☒	The statistical test(s) used AND whether they are one- or two-sided<br>*Only common tests should be described solely by name; describe more complex techniques in the Methods section.*
☐	☒	A description of all covariates tested
☐	☒	A description of any assumptions or corrections, such as tests of normality and adjustment for multiple comparisons
☐	☒	A full description of the statistical parameters including central tendency (e.g. means) or other basic estimates (e.g. regression coefficient) AND variation (e.g. standard deviation) or associated estimates of uncertainty (e.g. confidence intervals)
☐	☒	For null hypothesis testing, the test statistic (e.g. *F*, *t*, *r*) with confidence intervals, effect sizes, degrees of freedom and *P* value noted<br>*Give P values as exact values whenever suitable.*
☒	☐	For Bayesian analysis, information on the choice of priors and Markov chain Monte Carlo settings
☒	☐	For hierarchical and complex designs, identification of the appropriate level for tests and full reporting of outcomes
☒	☐	Estimates of effect sizes (e.g. Cohen's *d*, Pearson's *r*), indicating how they were calculated

Our web collection on statistics for biologists contains articles on many of the points above.

Software and code

Policy information about availability of computer code

Data collection	No software was used
Data analysis	Sequence annotation: StaG-mwc v.0.4.0 (fastp, Kraken2)<br>Diversity measurements: Qiime2 v.2020.2 (q2-diversity)<br>Statistical analyses: statsmodels v.0.11.1 (python 3.6.7), Seaborn v.0.1.0, Matplotlib v.3.1.3, R vegan v.2.5.6, scikit-bio v.0.5.6, q2-emperor<br>Differential abundance testing: pystan v.2.17.1.0 (songbird), DESeq2 v.1.30.1, ANCOM-II v2.1

For manuscripts utilizing custom algorithms or software that are central to the research but not yet described in published literature, software must be made available to editors and reviewers. We strongly encourage code deposition in a community repository (e.g. GitHub). See the Nature Portfolio guidelines for submitting code & software for further information.

Data

Policy information about availability of data

All manuscripts must include a data availability statement. This statement should provide the following information, where applicable:

- Accession codes, unique identifiers, or web links for publicly available datasets
- A description of any restrictions on data availability
- For clinical datasets or third party data, please ensure that the statement adheres to our policy

The data that support the findings of this study are openly available in the European Nucleotide Archive (ENA) database at https://www.ebi.ac.uk/metagenomics/, reference number [PRJEB52269].

Human research participants

Policy information about studies involving human research participants and Sex and Gender in Research.

Reporting on sex and gender	N/A
Population characteristics	N/A
Recruitment	N/A
Ethics oversight	N/A

Note that full information on the approval of the study protocol must also be provided in the manuscript.

Field-specific reporting

Please select the one below that is the best fit for your research. If you are not sure, read the appropriate sections before making your selection.

☒ Life sciences ☐ Behavioural & social sciences ☐ Ecological, evolutionary & environmental sciences

For a reference copy of the document with all sections, see nature.com/documents/nr-reporting-summary-flat.pdf

Life sciences study design

All studies must disclose on these points even when the disclosure is negative.

Sample size	Sample size N=64, distributed over each sex and condition (4) were acquired (n=8 per group). No sample-size calculation was performed. Number of samples was decided aiming to have substantial number of samples for robust statistical analysis, but few enough to follow animal handling ethics.
Data exclusions	One sample was excluded from the analysis that included adjustment for cages, because it lacked a cage control
Replication	Sequencing data acquired from animals could not be replicated therefore bigger (n>3, specifically 7 or 8 per group) were chosen to correct for variability. qPCR analyses were all performed in similar or larger numbers.
Randomization	Animals were allocated into experimental groups randomly, having the different experimental groups the same size and balanced between males and females within the groups.
Blinding	Blinding in this study was not relevant as it did not include assessments, the analysis bias was kept at minimum by extracting and processing the samples randomly and keeping animal metadata separately from the experimental data.

Reporting for specific materials, systems and methods

We require information from authors about some types of materials, experimental systems and methods used in many studies. Here, indicate whether each material, system or method listed is relevant to your study. If you are not sure if a list item applies to your research, read the appropriate section before selecting a response.

Materials & experimental systems

n/a	Involved in the study
☒	☐ Antibodies
☒	☐ Eukaryotic cell lines
☒	☐ Palaeontology and archaeology
☐	☒ Animals and other organisms
☒	☐ Clinical data
☒	☐ Dual use research of concern

Methods

n/a	Involved in the study
☒	☐ ChIP-seq
☒	☐ Flow cytometry
☒	☐ MRI-based neuroimaging

Animals and other research organisms

Policy information about studies involving animals; ARRIVE guidelines recommended for reporting animal research, and Sex and Gender in Research

Laboratory animals	Five- and six-week-old C57BL/6J mice of both sexes were obtained from in-house breeding.
Wild animals	Study did not involve wild animals
Reporting on sex	Sex was considered in the study design. Data was disaggregated for sex, using 32 males and 32 females total. All shown analyses were performed comparing sexes directly or by separating sexes before the analysis.
Field-collected samples	Study did not involve samples collected from the field.
Ethics oversight	The local ethical committee of the Swedish National Board of Animal Research (N230/15) approved all experimental protocols

Note that full information on the approval of the study protocol must also be provided in the manuscript.
